# Co-evolutionary pathways of synthetic biology regulation: A comparative analysis of major countries and Korea's strategic legislation

**DOI:** 10.71150/jm.2606027

**Published:** 2026-09-18

**Authors:** Hyeonsu Kim, Yeji Kwon, Heoung-yeol Kim, Jiyeon Lee

**Affiliations:** 1 National Biotechnology Policy Research Center, Korea Research Institute of Bioscience and Biotechnology (KRIBB), Daejeon 34141, Republic of Korea; 2 Graduate School of National Public Policy, Chungnam National University, Daejeon 34134, Republic of Korea

**Keywords:** biofoundry, co-evolution, institutional flexibility, preemptive legislation, regulatory lag, synthetic biology

## Abstract

The convergence of synthetic biology and artificial intelligence is widening the gap between technological advancement and existing regulatory frameworks — a phenomenon known as regulatory lag. As global competition intensifies to capture a synthetic biology market projected to reach approximately $70 billion by 2030, nations are responding to this challenge through markedly different approaches. This study applies a comparative legal methodology, drawing on co-evolutionary theory and the Law 1.0/2.0/3.0 framework, to analyze the regulatory responses of the United States, United Kingdom, Japan, and South Korea. The analysis reveals that national differences stem less from institutional design choices than from the triggers that initiate regulatory change, yielding four distinct co-evolutionary pathways. In the United States, a shift in the judicial environment prompted regulatory adaptation; in the United Kingdom, a political transition created the conditions for legislative reform; and in Japan, administrative reinterpretation of existing rules enabled regulatory adjustment. South Korea represents a fundamentally different case. By enacting the world’s first standalone synthetic biology legislation prior to full technological maturation, Korea has pursued a top-down co-evolutionary model in which institutional design precedes and shapes the trajectory of technological development. Three conclusions emerge from this analysis: there is no single optimal co-evolutionary pathway; institutional flexibility determines the sustainability of co-evolution; and co-evolution can itself be an object of proactive design. For Korea's preemptive model to prove effective, it requires a sufficient scientific foundation, institutional flexibility, and coherence with existing regulatory frameworks — conditions that offer a reference point for other nations confronting similar governance challenges.

## Introduction

The convergence of synthetic biology and artificial intelligence (AI) is emerging as a critical governance challenge, as the pace of technological advancement increasingly outstrips the capacity of existing regulatory frameworks (Jeong et al., 2023). This gap — commonly referred to as regulatory lag — creates a dual problem: it delays the market entry of innovative technologies while simultaneously leaving new risks in the blind spots of established oversight systems (Kim, 2017). The challenge is particularly acute in environments where AI autonomously designs genetic sequences by learning from large-scale biological data. In such contexts, the opacity of AI-driven design processes means that existing safety assessment frameworks, which were built around genetically modified organisms (GMOs), may not be fully equipped to address emerging risks (ETC Group, 2024). Microorganisms constitute the primary research objects of synthetic biology (Liu et al., 2020). Core areas of the field include the design of genetic circuits using microbial chassis such as *Escherichia coli* and yeast, the development of engineered strains through metabolic engineering, and high-throughput automated experimentation using biofoundries. The rapid advancement of such microorganism-based research has increasingly come into tension with existing GMO-centered regulatory frameworks, thereby exacerbating the problem of regulatory lag (Mandel and Marchant, 2014). The economic stakes of this governance gap are substantial. The global synthetic biology market is projected to reach approximately $70 billion by 2030 and $257.5 billion by 2036 (Future Markets Inc., 2025; Meige et al., 2024), with annual economic spillover effects estimated at $2–4 trillion across sectors including pharmaceuticals, healthcare, agriculture, and energy (McKinsey Global Institute, 2020). As expectations around this technological and economic potential grow, national investment in synthetic biology research and development has intensified accordingly. According to the Australian Strategic Policy Institute (ASPI), China produced approximately 60% of the world's highly cited synthetic biology publications between 2019 and 2023, rapidly closing the gap with the United States (Gaida et al., 2023). Against this backdrop, nations are pursuing diverse strategies to adapt or redesign their regulatory systems — a process that can be understood as co-evolution (Han, 2017), in which technology and institutions mutually shape one another over time. The ways in which countries respond to regulatory lag, however, are far from uniform. Some have adapted existing frameworks through changes in judicial interpretation; others have introduced new regulatory categories in response to political transitions; still others have recalibrated the scope of existing laws through administrative guidance. A fourth approach — enacting dedicated legislation and constructing public infrastructure in advance of full-scale commercialization — has also emerged as a distinct regulatory strategy. Existing research on synthetic biology regulation has largely focused on single-country regulatory analyses or the legal application of specific technologies. Comparative studies examining the triggers and pathways of regulatory change across countries through a co-evolutionary lens remain limited. To address this gap, this study compares the regulatory response pathways of the United States, the United Kingdom, Japan, and South Korea from a co-evolutionary perspective and examines the contextual conditions and distinctive features of each approach. In doing so, it seeks to explain the co-evolutionary dynamics of synthetic biology governance and to offer a comparative perspective on the design of legal and institutional frameworks in response to technological change.

## Theoretical Framework

### Concept of co-evolution

The co-evolution of technology and institutions can be understood as an interactive learning process in which the emergence of new technologies drives institutional change, and those institutional changes in turn shape the direction and conditions of further technological development (Han, 2017). Nelson’s concept of the co-evolution of technology, industrial structure, and supporting institutions (Nelson, 1994), alongside Freeman and Soete's innovation systems framework (Freeman and Soete, 1997), both demonstrate that technology and institutions do not stand in a linear, hierarchical relationship but instead dynamically shape and constitute one another. This study draws on this perspective as the theoretical foundation for analyzing synthetic biology regulatory governance. Within this framework, institutions simultaneously perform two functions. The first is regulation — controlling risks associated with new technologies. The second is promotion of innovation. When regulatory uncertainty is high or institutional frameworks are rigid, investment and research and development activities by innovative actors may be constrained (Bawa, 2011; Nature Materials, 2007). Conversely, when institutions actively fulfil their promotional function, law can serve as an instrument for designing national strategic objectives (Lee, 2016). Scholarly discussion of this technology-institution interaction has been extensive in the governance literature on emerging technologies such as nanotechnology and information and communications technology (Choung et al., 2006; Desierto, 2005; Song et al., 2006); however, comparative co-evolutionary analysis of synthetic biology regulation across multiple countries remains limited. In this study, co-evolution unfolds in two distinct directions. The first occurs when technological, judicial, or political change triggers the transformation of regulatory frameworks. The second arises when institutions take the lead in shaping the direction and conditions of technological development, thereby creating the institutional foundations for co-evolution. The former pattern is observed primarily in the cases of the United States, the United Kingdom, and Japan, whereas the latter is reflected in South Korea’s anticipatory approach, the practical effects of which remain to be assessed through its future implementation. This study therefore applies co-evolutionary theory to compare the regulatory response pathways of the United States, United Kingdom, Japan, and South Korea, and to examine how technology and regulation have been mutually adjusted within each country's distinctive institutional and political context.

### Literature review

Prior research on synthetic biology regulatory governance can be organized into five streams.

The first stream analyzes the co-evolution of technology and institutions from historical and comparative perspectives. Jasanoff (2005) empirically demonstrated that while biotechnology regulation in the United States, United Kingdom, and Germany each produced distinct civic epistemologies, these institutional settlements were ultimately destabilized as technology advanced (Jasanoff, 2005). This work establishes the methodological rationale for analyzing national regulatory responses through a co-evolutionary lens: once established, regulatory frameworks are not permanent but must be reconstituted in response to technological change.

The second stream theorizes the limitations of rigid regulation and advocates for adaptive governance. Mandel (2009) criticized the inflexibility of command-and-control regulatory approaches and called for a shift toward adaptive governance — a model in which regulatory frameworks are established early but revised iteratively as empirical evidence accumulates (Mandel, 2009). While this work provides a normative case for flexible governance, it does not extend to a concrete analysis of how different countries translate this principle into actual legal frameworks and policy instruments.

The third stream empirically documents the structural limitations of existing synthetic biology regulation. Sundaram et al. (2023) analyzed cases in which the European Union's binary regulatory framework left new biotechnology products in regulatory limbo, and argued for a spectrum-based approach better suited to the continuous nature of biological innovation (Sundaram et al., 2023). This work demonstrates that regulatory lag is not a product of policy failure in any particular country but rather a structural limitation of existing regulatory architectures. Separately, Undheim (2024) argued that in an environment where the convergence of AI and synthetic biology intensifies dual-use and biosecurity risks, ex post punitive regulation is insufficient to govern black-box innovation, and that proactive control at the infrastructure level is therefore necessary (Undheim, 2024). This argument provides direct support for the Safety by Design rationale associated with biofoundry-based governance, which is discussed further in the Discussion section.

The fourth stream theorizes collaborative governance between hard law and soft law instruments. Qin et al. (2026) identified structural limitations in traditional hard law frameworks — including the Biological Weapons Convention and the Convention on Biological Diversity — in addressing the technical characteristics of synthetic biology, and proposed a collaborative governance model characterized by a sequential dynamic: soft law provides proactive guidance, which is subsequently consolidated into hard law, with soft law continuing to incorporate feedback from operational experience (Qin et al., 2026). This framework offers theoretical grounding for the soft law operations and data feedback loop mechanisms associated with public biofoundry governance examined in this study.

The fifth stream typologizes the relationship between law and technology. Brownsword and Somsen (2021) proposed a three-stage framework — Law 1.0 (judicial interpretation), Law 2.0 (parliamentary legislation), and Law 3.0 (technology infrastructure-based regulation) — and argued that the transition to Law 3.0, in which technological systems themselves perform governance functions, is inevitable in an era of AI and automation (Brownsword and Somsen, 2021). This framework serves as the primary analytical typology for classifying national regulatory responses in the present study. Collectively, these bodies of work have made significant contributions to understanding the structural causes of regulatory lag and the theoretical case for adaptive governance. However, comparative legal analysis remains insufficient regarding the specific triggers through which regulatory change has been initiated in major countries and the distinct regulatory response pathways that have emerged as a result. This study seeks to address this gap through a comparative analysis of national regulatory change and response pathways.

### Analytical framework

To analyze the regulatory responses of each country, this study applies the Law 1.0/2.0/3.0 framework proposed by Brownsword and Somsen (2021), as reviewed above. This framework typologizes the relationship between law and technology into three stages — Law 1.0 (judicial interpretation), Law 2.0 (parliamentary legislation), and Law 3.0 (technology infrastructure-based regulation) — and argues that a transition toward Law 3.0, in which technological systems themselves perform governance functions, is inevitable in an era of AI and automation.

The three pathways serve as a useful analytical tool for typologizing how different countries respond to regulatory lag. Law 3.0 is particularly relevant in the context of synthetic biology increasingly integrated with AI and automation, as it highlights the possibility that infrastructure itself may perform safety management functions. This point is directly connected to the discussion of emerging governance challenges in the era of AI and automation.

The countries selected for comparison in this study are the United States, the United Kingdom, Japan, and South Korea. They were chosen according to three criteria. First, each country represents a distinct trigger of institutional change in synthetic biology governance, enabling a theoretically meaningful comparison. Second, all four possess their own legal and regulatory frameworks related to synthetic biology, as well as substantial research capabilities, thereby ensuring a reasonable degree of comparability. Third, primary sources—including legislation, administrative decisions, and judicial rulings—are sufficiently accessible for analysis. The European Union was excluded because regulatory authority over synthetic biology is dispersed across EU institutions and Member States, making it difficult to treat the EU as an analytical unit equivalent to a single country. China was also excluded because of limitations in the accessibility and transparency of primary regulatory materials. These exclusions are explicitly acknowledged in the Conclusion as limitations arising from the scope of this study. The comparative criterion across these cases is the trigger for regulatory change. The analytical premise of this study is that national differences stem not merely from choices in institutional design, but from the nature of the external shocks or internal drivers that initiated institutional change in each case.

## National Regulatory Pathways

### United States

The U.S. regulatory framework for synthetic biology was designed not around a single statute but as a distributed system in which multiple agencies share jurisdiction according to product characteristics. The 1986 Coordinated Framework for Regulation of Biotechnology established a flexible architecture in which the United States Department of Agriculture (USDA), the Food and Drug Administration (FDA), and the Environmental Protection Agency (EPA) each exercise authority under their respective existing statutory mandates (OSTP, 1986). The mechanism that allowed this system to function for decades was the Chevron doctrine, established in 1984 (Johnson et al., 2026). Whenever technologies such as gene editing or DNA synthesis emerged that did not exist at the time of enactment, administrative agencies could interpret and apply ambiguous statutory provisions with judicial deference under the Chevron doctrine (Johnson et al., 2026). In effect, the Chevron doctrine functioned as an institutional mechanism that enabled administrative agencies to interpret and fill gaps in existing statutes whenever new technologies emerged. This arrangement was fundamentally altered by the Supreme Court's 2024 decision in *Loper Bright Enterprises* v. *Raimondo*, which overruled the Chevron doctrine and required courts to exercise independent judgment in interpreting statutory ambiguity rather than deferring to administrative agencies (Choi, 2025). In the same year, the Supreme Court’s decision in *Corner Post* expanded the point from which the statute of limitations under the Administrative Procedure Act (APA) begins to run (Barczewski and Gaffney, 2024), creating an institutional environment in which even long-standing regulatory decisions may be challenged by newly affected parties (Barczewski and Gaffney, 2024). Concurrent reductions in federal agency staffing and budgets have further constrained the capacity of synthetic biology regulators to respond flexibly to new technologies without explicit statutory authorization (Haeder, 2025). Against this backdrop, the National Security Commission on Emerging Biotechnology (NSCEB) observed that the *Loper Bright* decision had narrowed administrative agencies’ regulatory discretion and heightened regulatory uncertainty in the field of synthetic biology (NSCEB, 2026). It accordingly proposed establishing a National Biotechnology Coordination Office (NBCO), together with a regulatory sandbox model under which common platforms, such as chassis microorganisms, would be pre-certified and the review of derivative products streamlined. These recommendations were subsequently incorporated into proposals contained in the *National Biotechnology Initiative Act of 2025* (H.R. 2756), introduced in Congress in April 2025, which proposes the creation of the NBCO and the development of an interagency regulatory coordination framework (United States Congress, 2025). This case illustrates how an external shock arising from a change in the judicial environment triggered the redesign of the regulatory system. While it is an observed fact that institutional change is currently underway, the practical effects of this transition on the synthetic biology research and development ecosystem are expected to become clearer only after the legislative process has been completed.

### United Kingdom

The starting point for the United Kingdom case is the structural rigidity of the European Union's GMO regulatory framework. EU GMO regulation, established in the 1990s, focused on the technological process rather than the product, and in 2018 the Court of Justice of the European Union (CJEU) ruled that products of precision breeding techniques such as CRISPR must be regulated identically to conventional GMOs (CJEU, 2018). This ruling intensified debate over authorization costs and review timelines, and the scientific community continued to press for a regulatory framework more commensurate with the technical characteristics of precision breeding. Under EU membership, however, institutionalizing this demand was not possible. Brexit served as the turning point that altered this situation. Departure from the EU regulatory system opened space for the United Kingdom to design an independent framework, resulting in the enactment of *The Genetic Technology* (*Precision Breeding*) *Act 2023* (*DEFRA, 2023*). The Act's central innovation was the creation of a new legal category — Precision Bred Organisms (PBOs) — to distinguish gene-edited products that could have arisen through conventional breeding or natural mutation from products regulated as conventional GMOs (Watson and Hayta, 2024). Based on scientific advice from the Advisory Committee on Releases to the Environment (ACRE), PBOs became subject to a risk-proportionate, streamlined authorization procedure, with regulatory review timelines projected to shorten from a maximum of approximately ten years to approximately twelve months (DEFRA, 2022). Procedurally, the Act shifted from prior authorization to a notification-based system in which developers self-verify PBO eligibility before notifying regulators, centering oversight on post-market monitoring rather than pre-market approval. This institutional transition was operationalized through *The Genetic Technology* (*Precision Breeding*) *Regulations 2025*, which specified the procedures for PBO eligibility verification (UK Statutory Instruments, 2025), the marketing notification system, and the criteria for food and feed safety assessment. Practical implementation has followed: in April 2026, gene-edited barley developed by Rothamsted Research received the United Kingdom's first PBO marketing authorization under this framework (DEFRA, 2026), demonstrating that the Precision Breeding Act has moved beyond legislative declaration to function as an institutional foundation supporting the commercialization of innovative products. Even after the CJEU ruling, regulatory restructuring remained difficult to achieve within the EU framework, whereas Brexit created the political space necessary for the United Kingdom to design an independent regulatory system. This suggests that scientific necessity may serve as a driver of regulatory reform, but that actual institutional change becomes possible only when it is supported by enabling political conditions. The enactment of the Precision Breeding Act also illustrates how regulatory change can redefine the conditions for technological development. The United Kingdom’s first PBO marketing authorization in April 2026 constitutes an observed outcome in which institutional change translated into the market entry of a technological product.

### Japan

The Japanese case represents a pathway of administrative adaptation — one in which new technologies were addressed not through new legislation or judicial change, but through scientific reinterpretation of existing law and administrative guidance. The foundational statute for synthetic biology regulation in Japan is the Cartagena Act, which defines living modified organisms (LMOs) as organisms obtained by transferring nucleic acids processed outside the cell into a cell (Government of Japan, 2003). As genome editing technologies such as CRISPR emerged, the scope of this definition became a central regulatory question, and the Japanese government chose to respond through scientific interpretation rather than new legislation.

In February 2019, the Ministry of the Environment (MOE) and relevant ministries classified genome editing techniques into three categories — SDN-1, SDN-2, and SDN-3 — and officially determined that SDN-1 methods, which do not use DNA templates and leave no foreign nucleic acid in the final product, do not fall within the definition of LMOs under the Cartagena Act (Tsuda et al., 2019). Organisms produced using SDN-2 and SDN-3 methods, which involve the insertion of foreign nucleic acids, were classified as LMOs and brought within the regulatory scope; however, products meeting the criteria for self-cloning or natural occurrence were exempted from regulation in the same manner as under existing LMO policy (Kim et al., 2018). This approach recalibrated the regulatory boundary not by constructing a new framework for genome editing but by applying the principles of existing LMO policy consistently to the new technical context.

The Japanese government also established a separate administrative management system for SDN-1 products, introducing administrative guidance incorporating a pre-submission consultation and notification procedure to secure transparency and public trust (MEXT, 2025). Information on genetic changes, functions, and biodiversity impacts of notified products was made publicly accessible through a dedicated website (Kondo and Taguchi, 2022), enabling stakeholder access to relevant data. As a practical outcome of this approach, genome-edited food products — including the Sicilian Rouge High GABA Tomato, engineered to accumulate elevated levels of γ-aminobutyric acid (GABA) — have reached the market through this regulatory pathway (MHLW, 2021).

These administrative responses enhanced the clarity and predictability of regulatory application to genome-edited organisms in Japan and provided a reference point for OECD discussions and the regulatory deliberations of other countries (Tsuda et al., 2019). Japan’s experience demonstrates that regulatory frameworks for emerging technologies can be adjusted through science-based administrative interpretation without enacting new legislation. The decision to exempt SDN-1 applications from existing GMO regulations also reduced the regulatory burden on research and development using genome-editing technologies, producing the observed outcome of enabling genome-edited foods, such as high-GABA tomatoes, to enter the market. This can be understood as a case in which institutional change substantively reshaped the conditions for technological development.

For this approach to function stably, however, consistency in administrative interpretation, transparency in decisional criteria, and public accessibility of relevant information are essential supporting conditions.

## Korea: Proactive Co-Evolution

### Background: Scientific foundations and policy trajectory

Korea's proactive legislative approach can be understood not as an isolated policy decision but as the product of a gradual convergence between accumulated scientific and technological capability and emerging national strategic priorities. In terms of scientific foundations, Korea published a total of 1,341 synthetic biology papers between 2015 and 2024, ranking seventh globally in output (Kim and Lee, 2025). More significant than volume, however, is the quality of that research. Korea's mean Relative Citation Ratio (RCR) — a field-normalized measure of research influence — averaged 1.84 over the past decade, placing it sixth globally, and reached 1.99 in 2023, ranking third worldwide. These figures indicate that Korea has developed internationally competitive research capacity in synthetic biology even before entering the stage of full-scale industrialization. Given that institutional frameworks established without sufficient scientific foundations risk becoming ineffective in practice, this research performance served as one of the substantive bases legitimizing proactive legislation.

The policy dimension reflects an equally deliberate process of institutional accumulation. The Korean government designated advanced biotechnology as a national strategic technology in October 2022, followed in December of the same year by the announcement of the National Synthetic Biology Promotion Strategy (MSIT, 2022). In 2024, the Strategy for Development and Diffusion of Core Synthetic Biology Technologies further specified six priority domains — including biomolecular design and DNA/RNA fabrication — along with medium- and long-term development directions (MSIT, 2024b). In 2024, a public biofoundry infrastructure development project valued at approximately 126.3 billion Korean won passed a preliminary feasibility assessment (MSIT, 2024a). The sequential progression (Fig. 1) from strategic declaration to infrastructure investment, and from infrastructure investment to legislation, suggests that the resulting Act should be understood not as the terminal point of a policy process but as the legal formalization of a national strategy already substantially underway.

### Legal formalization of strategic technology

As technology development and infrastructure investment intensified, Korea recognized the need for an institutional foundation to secure core technologies in synthetic biology and to respond to intensifying global competition over technological leadership. From this perspective, the *Synthetic Biology Promotion Act* represents a form of strategic legislation — one through which the state proactively designs the direction and conditions of technological development rather than merely a reorganization of existing regulatory arrangements (Kim, 2024a). The sequence of policy actions reviewed in the preceding section — the designation of advanced biotechnology as a national strategic technology, the establishment of the National Synthetic Biology Promotion Strategy, and the passage of the public biofoundry construction project through preliminary feasibility assessment — shows that the Act institutionalized a national strategy already in progress, rather than initiating a wholly new policy direction.

This approach is distinguishable from the cases of the United States, United Kingdom, and Japan. Whereas those three countries adjusted existing institutional frameworks in reaction to a judicial shift, a political transition, or the need for administrative reinterpretation, Korea chose to enact new standalone legislation governing synthetic biology before external regulatory pressures or institutional shocks had fully materialized. This constitutes a top-down co-evolutionary approach in which institutional design precedes and shapes the trajectory of technological development, rather than reactively responding to regulatory lag. In the Korean case, institutions have proactively shaped the direction and conditions of technological development, with institutional design preceding full technological maturation rather than technological advancement serving as the primary driver of institutional change.

### Key features of the Synthetic Biology Promotion Act

Following legislative deliberations that began in 2023 (National Assembly of the Republic of Korea, 2023, 2024), the *Synthetic Biology Promotion Act* was enacted in April 2025, comprising six chapters and thirty articles. The Act is the world’s first statute to incorporate into its statutory structure the five principles set out in the OECD’s *Framework for Anticipatory Governance of Emerging Technologies* (2024): guiding values, strategic intelligence, stakeholder engagement, agile regulation, and international co-operation (OECD, 2026). Its defining characteristic is not the introduction of new regulations targeting specific technologies or products, but rather the institutionalization of the entire synthetic biology research and development ecosystem. Whereas prior biotechnology legislation focused primarily on hazard management of final products or the regulation of individual research activities, this Act (Table 1) establishes in statutory form the core infrastructure spanning the full research and development cycle — including public biofoundries, research data, and safety management systems. This institutional design can be understood through three pillars (Lee, 2025). The first pillar is the statutory entrenchment of infrastructure. By specifying the public biofoundry in legislation, the Act establishes the legal basis for open access by industry, academia, and the research community (Article 19). Anchoring infrastructure in statute rather than in policy programs carries the institutional significance of providing an institutional basis for continuity despite changes in government or fluctuations in budget allocations. The second pillar is data governance. The Act establishes shared data use rights between research entities and operating institutions, while requiring the prior consent of the research entity as a condition for third-party support, thereby seeking to balance data utilization with the protection of intellectual property rights (Article 20). The third pillar is the proactive internalization of safety. By specifying research and development guidelines and a safety management system, the Act is designed to embed safety management from the earliest stages of the research process — a departure from the existing Act on Living Modified Organisms (LMO Act), which focused primarily on the ex post management of risks associated with final products (Articles 25 and 26).

The unifying principle across these three pillars is the integration of promotion and regulation. A public survey conducted during the legislative process found that 41.7% of respondents identified technology promotion as a priority, while 35.3% identified risk management as a priority (Kim, 2024b) — and the Act's attempt to accommodate both imperatives within a single framework can be interpreted as broadly consonant with the concept of "prudent vigilance" in synthetic biology governance, which seeks to pursue innovation enablement and risk management simultaneously (Cho, 2022). The Act also anticipates a structural tension that is likely to become visible once the public biofoundry enters full operation: the case-by-case approval system under the existing LMO Act is structurally incompatible with the high-throughput, automated experimental cycles characteristic of biofoundry operations. This incompatibility may generate pressure for regulatory reform, and the role of the proactive legislation lies not in directly reforming that regulatory framework but in securing in advance the mechanism through which the operational realities of biofoundry activity can drive such change.

## Discussion

### Comparing four co-evolutionary pathways

The comparative analysis of four national cases (Table 2) yields three implications for understanding how synthetic biology governance operates.

The first is that co-evolution does not follow a single universal pathway but takes on distinct forms shaped by each country's institutional and political context. The United States responded to a shift in its judicial environment; the United Kingdom restructured its regulatory framework in response to a political transition; and Japan addressed new technologies through administrative reinterpretation of existing law. These cases suggest that co-evolution is not governed by a universal model but is instead constituted by the particular institutional conditions and policy environments of each country. The purpose of comparative analysis is therefore not to identify a single best practice but to understand the conditions under which each pathway functions.

The second implication concerns the relationship between institutional flexibility and the sustainability of co-evolution. The United States case suggests that heavy reliance on a single regulatory mechanism — such as the Chevron doctrine — can generate systemic uncertainty across the regulatory framework when that mechanism is altered by judicial change. Japan's approach through administrative interpretation offers flexibility but lacks legal stability, while the United Kingdom's legislation-centered approach provides stability but remains susceptible to shifts in the political environment. Taken together, these cases suggest that excessive dependence on any particular institutional instrument can itself introduce new sources of uncertainty in the process of responding to technological change.

The third implication is that co-evolution need not be confined to reactive adaptation following technological change but can itself be an object of proactive institutional design. Whereas the cases of the United States, the United Kingdom, and Japan primarily illustrate processes of adaptation to external environmental change, South Korea institutionalized research ecosystem infrastructure—such as public biofoundries and data governance—before undertaking direct regulatory reform. The adoption of this institutional design is itself an observed fact. Whether it will lead to the actual adaptation and improvement of the regulatory system, however, remains to be assessed through the implementation of the Act. This case may extend existing co-evolutionary theory, which has predominantly conceptualized technology–institution co-evolution as a process of mutual adaptation, by demonstrating that institutions can proactively shape the conditions for co-evolution even at a stage when the technology has not yet fully matured.

These three implications extend into a more complex set of governance challenges in the technological context of AI and automation. The possibility of a transition toward infrastructure itself performing governance functions — viewed through the lens of Law 3.0 — is examined in the following section, with particular attention to the role of biofoundries.

### Governance challenges in the era of AI and automation

The convergence of AI and automation with synthetic biology structurally intensifies the triggers for regulatory change. In an environment where AI systems trained on large-scale biological data automate genetic design, Law 2.0 approaches based on parliamentary legislation alone are unlikely to keep pace with the speed of technological development. The alternative that has attracted growing attention is a Law 3.0 approach, in which technological infrastructure itself performs governance functions. Biofoundries — platforms that conduct the full research and development cycle of synthetic biology within a standardized, automated environment — are regarded as a potential vehicle for realizing this approach. Korea's statutory specification of the public biofoundry through Article 19 of the *Synthetic Biology Promotion Act* can be interpreted as an institutional acceptance of this direction.

Public biofoundries hold the potential to perform governance functions across three dimensions. The first is Safety by Design. If AI-based screening systems within a biofoundry are designed to detect and block potentially hazardous genetic sequences at an early stage, safety management becomes embedded in the research process itself rather than operating as ex post regulation (Kim et al., 2025). In particular, if the biosafety of engineered microorganisms can be assessed at the design stage during microbial chassis-based strain engineering using organisms such as *Escherichia coli* and yeast, this would constitute a fundamentally different approach from the ex post risk assessment applied under the existing LMO Act. The second is research data governance. Data accumulated through biofoundry operations are simultaneously a source of innovation and a potential subject of intellectual property disputes. Article 20 of the *Synthetic Biology Promotion Act* seeks to balance data utilization with rights protection by requiring the prior consent of the research entity when data are used to support a third party (McLennan and Maslen, 2025). The third is pre-certification-based regulatory streamlining. Analogous to the FDA's Digital Health Pre-Cert model, a system in which an integrated framework encompassing both infrastructure and operations is certified in advance — with individual research activities subject to simplified administrative review — represents a plausible medium- to long-term direction for biofoundry regulation (Lievevrouw et al., 2022).

It should be noted, however, that these functions represent a potential trajectory to be realized incrementally through future institutional design, rather than capabilities already possessed by Korea's public biofoundry. Accordingly, the discussion of Law 3.0 governance presented in this study should be understood not as a description of the current state of governance, but as a direction for future policy design. Should this foundation become linked to international standardization discussions in due course, the possibility also exists that Korea could function as a norm-setter in global regulatory harmonization.

### Conditions and limitations of the proactive co-evolutionary model

Three conditions must be met for Korea's proactive co-evolutionary model to prove effective in practice.

The first is a sufficient scientific foundation. Institutions can be designed ahead of technology, but where no technological substance exists, institutions become hollow. In the Korean case, research performance ranking sixth to seventh globally served as one of the important foundations supporting proactive legislation. Conversely, where institutions are established without a corresponding accumulation of research capacity, legislation risks remaining a declaration disconnected from practice.

The second condition is institutional flexibility. When subordinate regulations are designed in excessive detail at a stage when the technology has not yet matured, a paradox can emerge in which institutions themselves constrain innovation — what might be termed the paradox of proactive regulation. This paradox represents a realistic challenge the *Synthetic Biology Promotion Act* may face. One approach worth considering is to confine the statute itself to the level of declaring basic principles, while operating specific safety management standards in the form of soft law— adjustable to circumstances, along the lines of Japan's pre-submission consultation system (MEXT, 2025) or the United States' regulatory sandbox model (NSCEB, 2026). It is also worth considering the incorporation of a periodic mechanism into the statutory framework to reassess technological maturity and regulatory suitability.

The third condition is coherence with existing regulatory frameworks. The *Synthetic Biology Promotion Act* is a promotion-oriented statute administered by the Ministry of Science and ICT, while the LMO Act is a safety management statute under the joint jurisdiction of multiple ministries including the Ministry of Trade, Industry and Energy and the Ministry of Agriculture, Food and Rural Affairs. In areas where the scope of the two statutes overlaps, coordination of ministerial roles and standards will be necessary — a task that remains to be addressed incrementally through implementing legislation and cross-ministerial governance arrangements.

These considerations are not confined to the Korean case and may serve as reference points for other countries considering similar approaches. Proactive institutional design is better understood as a starting point for co-evolution than as its completion; its effectiveness may be enhanced when supported by the sustained capacity to review and refine institutional arrangements in response to technological change.

## Conclusion

This study analyzed the problem of regulatory lag in synthetic biology governance through a co-evolutionary framework and typologized the regulatory responses of the United States, the United Kingdom, Japan, and South Korea into four distinct pathways. A shift in the judicial environment, a political transition, the need for technical reinterpretation, and a deliberate national strategic choice served as the respective triggers for institutional change in each country — demonstrating that co-evolution does not unfold according to a single universal pattern.

This analysis makes two scholarly contributions. First, existing co-evolutionary theory has predominantly conceptualized institutional change as a reactive and incremental process of adaptation to technological development. This study shows that the cases of the United States, United Kingdom, and Japan each represent reactive responses to external shocks, while simultaneously demonstrating through the Korean case that states can proactively shape the conditions for co-evolution by proactively designing research ecosystem infrastructure. This constitutes a complementary contribution that extends the typology of co-evolutionary theory. Second, the co-evolutionary framework developed here is not confined to synthetic biology; it is applicable to governance research on other emerging technologies — including AI and quantum technology — where regulatory lag arises as a structural feature. Taken together, these contributions extend existing scholarship that has understood co-evolution primarily as a process of reactive adaptation to technological change, while offering the analytical categories of regulatory triggers and regulatory pathways as tools for comparative research on emerging technology governance.

Three policy implications follow from this analysis. First, regardless of which pathway a country follows, excessive dependence on any single institutional instrument should be avoided. Second, in an era of AI and automation, it is necessary to prepare infrastructure-based Law 3.0 governance as a medium- to long-term direction. Third, in the Korean case, if the jurisdictional overlap between the *Synthetic Biology Promotion Act* and the LMO Act cannot be resolved through cross-ministerial governance, the effects of proactive legislation risk being offset by institutional fragmentation — a challenge that is equally relevant to other countries pursuing similar approaches. These three implications are not independent of one another: diversification of institutional instruments, infrastructure-based governance, and cross-ministerial coherence can be understood as jointly determining conditions for the sustainability of the proactive co-evolutionary model.

To maintain consistency in the unit of analysis, this study does not include the European Union or China. The EU is difficult to treat as an analytical unit equivalent to a single country because regulatory authority over synthetic biology is distributed among Member States. China was excluded because of limitations in the accessibility of primary regulatory materials. The exclusion of other major actors, including Australia, and the limited treatment of actual implementation and enforcement processes also remain important limitations of the study’s scope. Future research should expand the comparative case base, conduct longitudinal case studies tracking the implementation of Korea's *Synthetic Biology Promotion Act*, and pursue cross-case analysis with other emerging technologies — to test whether proactive co-evolution represents an exceptional case or a replicable model.

## Figures and Tables

**Fig. 1.**
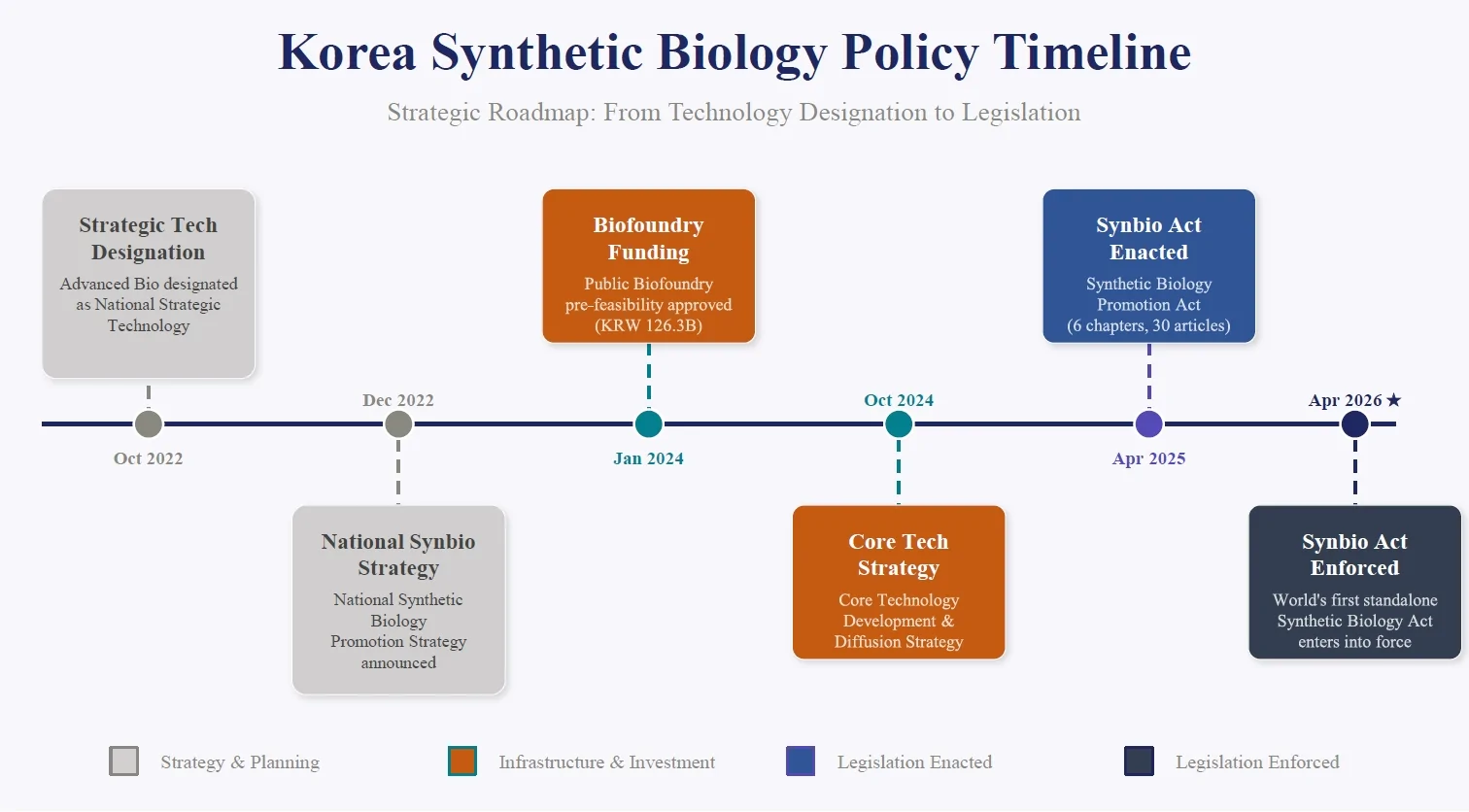
Korea's synthetic biology policy timeline from strategic designation to legislative enforcement (2022–2026). Sequential progression of national policy milestones across three phases: strategy and planning (gray), infrastructure and investment (orange), and legislation (blue). Strategic Tech Designation, Oct 2022: designation of advanced biotechnology as a national strategic technology. National Synbio Strategy, Dec 2022: announcement of the National Synthetic Biology Promotion Strategy. Biofoundry Funding, Jan 2024: preliminary feasibility approval for the public biofoundry construction project (KRW 126.3 billion). Core Tech Strategy, Oct 2024: announcement of the Core Technology Development and Diffusion Strategy. Synbio Act Enacted, Apr 2025: enactment of the *Synthetic Biology Promotion Act* (6 chapters, 30 articles). Synbio Act Enforced, Apr 2026: entry into force of the world's first standalone synthetic biology statute.

**Table 1.** Structure of the Synthetic Biology Promotion Act (6 chapters, 30 articles)

**Chapter 1. General Provisions**	
Art. 1	Purpose: securing foundational stability
Art. 2	Definitions of synthetic biology
Art. 3	State obligations
Art. 4	Relationship with other statutes
**Chapter 2. Promotion and Governance**	
Art. 5	Basic plan (every 5 years)
Art. 6	Annual implementation plan
Art. 7	Synthetic Biology Working Committee
Art. 8	Recommendations for regulatory improvement
Art. 9	Domestic and international landscape survey
Art. 10	Statistics compilation and management
Art. 11	Technology impact and level assessment
Art. 12	Synthetic Biology Development Council
Art. 13	Designation of policy specialist institution
**Chapter 3. R&D Promotion and Support**	
Art. 14	R&D programs and technology roadmap
Art. 15	Designation of R&D hub institutions
Art. 16	Innovation support for industry, universities, and research institutes
Art. 17	Technology transfer and commercialization
**Chapter 4. Research Infrastructure**	
Art. 18	Support for research facilities and equipment
Art. 19	Establishment of public biofoundry
Art. 20	Shared use of research data
Art. 21	Research data utilization policy
Art. 22	Synthetic biology standardization
Art. 23	Workforce development
Art. 24	International cooperation
**Chapter 5. Responsible Management**	
Art. 25	R&D guidelines
Art. 26	Safety management system
Art. 27	Stakeholder consultation
Art. 28	Public understanding and outreach
**Chapter 6. Supplementary Provisions**	
Art. 29	Delegation and entrustment of authority
Art. 30	Civil servant status for penalty purposes

**Table 2.** Four co-evolutionary pathways in synthetic biology governance across selected countries

Country	Trigger for regulatory change	Key regulatory response
United States	Shift in judicial environment	Introduction of H.R. 2756 (*National Biotechnology Initiative Act of 2025*)
United Kingdom	Political transition	Enactment of the Genetic Technology (*Precision Breeding*) Act 2023
Japan	Need for technical reinterpretation	SDN classification and parallel soft law mechanisms
South Korea	Proactive institutional design	Enactment of the *Synthetic Biology Promotion Act*
